# Germline Mutation in *KIF1Bβ* Gene Associated with Loss of Heterozygosity: Usefulness of Next-Generation Sequencing in the Genetic Screening of Patients with Pheochromocytoma

**DOI:** 10.1155/2020/3671396

**Published:** 2020-05-30

**Authors:** Giuseppina De Filpo, Elisa Contini, Viola Serio, Andrea Valeri, Massimiliano Chetta, Daniele Guasti, Daniele Bani, Massimo Mannelli, Elena Rapizzi, Michaela Luconi, Mario Maggi, Tonino Ercolino, Letizia Canu

**Affiliations:** ^1^Dept. of Experimental and Clinical Biomedical Sciences, University of Florence, Florence, Italy; ^2^Endocrinology Unit, Azienda Ospedaliero-Universitaria Careggi, Florence, Italy; ^3^Center of Research and Innovation of Myeloproliferative Neoplasms, AOU Careggi, University of Florence, Florence, Italy; ^4^General and Surgical Unit, Azienda Ospedaliero-Universitaria Careggi, Florence, Italy; ^5^A.O.R.N. Cardarelli, Medical Genetics, Pad. Y, Naples, Italy; ^6^Department of Experimental and Clinical Medicine, University of Florence, Florence, Italy

## Abstract

The genetic approach of pheochromocytomas and paragangliomas has changed in the last two decades. Nowadays, we know that more than 40% of patients have a germline mutation in one of the susceptibility genes identified to date. Our aim is to underline how genetic diagnosis by next-generation sequencing (NGS) can improve the management of patients affected by pheochromocytomas and paragangliomas in our routine diagnostic screening. We reported a case presentation and next-generation sequencing analysis supported by in silico studies and evaluation of mitochondrial status in *KIF1Bβ* tissue. A 46-year-old male affected by a left secreting pheochromocytoma underwent surgery in 2017. After surgery, the normetanephrine levels decreased very slowly and a suspected abdominal lymph node was detected. We found a novel germline *KIF1Bβ* gene mutation, c.4052C > T, p. Pro1351Leu associated with tumor loss of heterozygosity, and resulted likely-pathogenetic by in silico studies. This mutation was also associated with an increased number of mitochondria through the electron microscopy compared with wild-type tissues as suggestive for mitochondria neoformation compensatory to the mitochondrial autophagic figures observed. Our results underline the usefulness of next-generation sequencing in the presence of multiple tumor predisposition genes and how, at the same time, its use may result challenging for the clinicians. To date, performing the genetic analysis according to the latest Consensus Statement is mandatory in patients affected by PHEO/PGL.

## 1. Introduction

Pheochromocytomas (PHEO) and paragangliomas (PGL) are rare tumors with an incidence around 2–5 patients per million per year. In 2017, the WHO Classification of Tumors of Endocrine Organs acknowledged this unpredictable behavior classifying PHEO and PGL among malignant category (ICD-O/3) [1]. Up to 40% of PHEO/PGL are caused by germline mutations in one of the current 14 main known susceptibility genes [2]. Moreover, mutations of these and other genes have been found at the somatic level, in tumor tissue. In the past, the search for germline mutations in patients with PHEO/PGL was clinically driven [3] but, with the advent of the next-generation sequencing (NGS), targeted panels have been created allowing the simultaneous analysis of the susceptibility genes, according to the Consensus Statement on the genetic diagnosis of PHEO/PGL [4].

In particular, we test simultaneously many susceptibility genes: *EGLN1, EPAS1, FH, KIF1Bβ, MAX, NF1, RET, SDHA, SDHAF2, SDHB, SDHC, SDHD, TMEM127,* and *VHL*. During the last two decades, knowledge of the genetic basis of PHEO and PGL has undergone important advances. Transcriptome studies divided PHEO/PGL into two main clusters: cluster 1 included *PHD2*-, *VHL*-, *SDHx*-, *IDH*-, *HIF2A*-, *MDH2*-, and *FH*-mutated tumors while cluster 2 included *RET*-, *MAX*-, *NF1*-, *TMEM127*-, and *KIF1Bβ*-mutated tumors plus sporadic PHEO/PGL [5].

*KIF1B* is a member of the kinesin 3 family genes with a specific cellular role in energy transport. The gene is located at chromosome 1p36.22 and encodes two isoforms, *KIF1Bα* and *KIF1Bβ*, the latter acting as a tumor suppressor gene necessary for neuronal apoptosis [6]. A *KIF1Bβ* germline mutation was described in a family affected by Charcot-Marie-Tooth (CMT) type 2A [7].

Li et al. [8] demonstrated that *KIF1Bβ* controls mitochondrial fission through the activation of calcineurin (CN) implicated in the dephosphorylation of dynamin-related protein (DRP1) at Ser636 position and that the translocation of DRP1 from the cytoplasm to the mitochondria results in mitochondrial fission and apoptosis. Also Ando et al. [9] reported the central role of *KIF1Bβ* in the mitochondria-mediated apoptosis; in particular, they demonstrated that *KIF1Bβ* overexpression induces mitochondrial fragmentation and apoptosis interacting with a mitochondrial metalloprotease, YME1L1.

*KIF1Bβ* was identified as a susceptibility gene for neuroendocrine diseases about ten years ago, but, to date, only a few variants have been associated with cluster 2 PHEO/PGL [10, 11].

## 2. Materials and Methods

### 2.1. Clinical Report

A 46-year-old male affected by a left incidentally detected PHEO was referred to our unit in 2017. A computed tomography (CT) scan and a magnetic resonance imaging (MRI) confirmed the presence of a solid lesion in the left adrenal gland, 8.5 cm in size, with absolute and relative washout of 20% and 10%, respectively, at enhanced CT scan.

The urine test showed high levels of urinary metanephrine (MNu: 4033 mcg/24 h, normal value (nv) < 320) and urinary normetanephrine (NMNu: 5234 mcg/24h, nv < 390), confirming the presence of a PHEO. At the patient's history, there was no evidence of tumor risk factors.

The patient underwent surgery in November 2017; the surgical course was uneventful, and the histological examination confirmed the presence of a left PHEO. At pathology report, Ki67% was <1% and necrosis and vascular invasion were absent. Pheochromocytoma of Adrenal gland Scaled Score (PASS) [12] was not reported.

The patient gave his written informed consent to genetic testing, and all the major susceptibility genes were assayed (*EGLN1, EPAS1, FH, KIF1Bβ, MAX, NF1, RET, SDHA, SDHAF2, SDHB, SDHC, SDHD, TMEM127,* and *VHL*) by NGS. Only a variant of uncertain significance (*VUS*) in exon 38 of *KIF1Bβ* was found.

One month after surgery, the levels of urinary metanephrines were still elevated (MNu 118 mcg/24 h; NMNu 946 mcg/24 h). Subsequent controls confirmed the increase in NMNu up to 1026 mcg/24 h in March 2018. The patient performed an abdominal MRI which revealed the presence of an enlarged lymph-node, close to the area of surgery, that showed a weak positive uptake at ^123^I-metaiodobenzylguanidine (^123^I-MIBG) scintigraphy and at ^18^F-fluoro-dihydroxyphenylalanine (^18^F-DOPA) positron emission tomography (PET).

At the latest control, after two years from surgery, we observed a normal level of NMNu (389 mcg/day). A control abdominal MRI has been planned to evaluate the state of the abdominal lymph-node.

### 2.2. Molecular Analysis

After obtaining informed consent, genomic DNA of the patient was extracted by the QIAsynfony CDN kit (Qiagen). DNA quality and quantity were measured by the Qubit ds assay on the Qubit 2.0 fluorometer (Thermo Fisher Scientific).

A panel of 14 genes was designed using the online Sure Design software (Agilent Technologies). A predesigned Haloplex Pheochromocytoma panel containing the genes *EGLN1, EPAS1, FH, KIF1Bβ, MAX, NF1, RET, SDHA, SDHAF2, SDHB, SDHC, SDHD, TMEM127,* and *VHL* was used. The size of the final target region was 38.813 kpb with 2170 amplicons, and the mean sequence coverage was 98.53 at 20X priori 99.41% coverage of the target region.

Libraries were generated using the Agilent HaloPlex Target Enrichment protocol, according to the manufacturer's instructions and sequenced as 150 bp paired-end reads on the Illumina Miseq platform.

Reads quality was checked with FastQC (http://www.bioinformatics.babraham.ac.uk/projects/fastqc), and reads were aligned to the reference human genome hg19 with BWA-MEM alignment [13]. Genome Analysis Toolkit (GATK) [14] was used to recalibrate base qualities and realign aligned reads around insertion/deletions (InDels).

Finally single-nucleotide variants (SNVs) and InDels were identified by the Unified Genotyper module of GATK and functional annotation by ANNOVAR [15].

BASH and R custom scripts based on BedTools CoverageBed analysis [16] were used to obtain the list of low coverage (≤20X) regions per sample.

Clinically relevant variants have been classified according to the American College of Medical Genetics and Genomics and the Association for Molecular Pathology (ACMG) guidelines [17].

### 2.3. Loss of Heterozygosity (LOH) Analysis

To determine whether there was LOH at the mutation position (c.4052C > T), we undertook sequence analysis of *KIF1Bβ* exon 38 and flanking intronic regions (NM_003002.2) in tumor DNA from the tissue removed at surgery, with standard PCR amplification. PCR products were sequenced by standard direct sequencing with a BigDye version1.1 kit (Applied Biosystems, Foster City, California). Sequencing reactions were analyzed using a model 310 ABI PRISMA genetic analyzer, and the data were processed by sequencing analysis (Applied Biosystems, Foster City, California).

### 2.4. Three-Dimensional (3D) Mutation Prediction

*KIF1Bβ* 3D structures were modeled using I-TASSER (Iterative Threading ASSEmbly Refinement https://zhanglab.ccmb.med.umich.edu/I-TASSER/), a hierarchical approach to protein structure and function prediction. The analysis provides structural templates from the Protein Data Bank (PDB). Function insights of the target are then defined by COACH (https://zhanglab.ccmb.med.umich.edu/COACH/) to predict protein-ligand binding site [18–20].

The ^*∗*^ pdb files generated from I-TASSER were loaded and visualized with ChemDraw software (version 8; Cambridge Software; PerkinElmer, Inc., Waltham, MA, USA).

### 2.5. Electron Microscopy

Tissue specimens (2 × 2 mm) were washed with PBS and were directly fixed in Karnovsky (cold 2.5% glutaraldehyde and 2% formaldehyde) in 0.1 M sodium cacodylate buffer (pH 7.4) overnight at 4°C and postfixed in cold 1% osmium tetroxide in 0.1 M phosphate buffer (pH 7.4) for 1h at room temperature. The samples were dehydrated in graded acetone, passed through propylene oxide, and embedded in epoxy resin. Ultrathin sections were stained with gadolinium acetate and alkaline bismuth subnitrate and examined under a JEM 1010 electron microscope (Jeol, Tokyo, Japan) at 80 kV. Photomicrographs were taken with a MegaView III (Soft Imaging System, Muenster, Germany) digital camera connected with a personal computer with dedicated software (AnalySIS, Soft Imaging Software, Muenster, Germany) [21]. Results of electron microscopy studies were compared with those obtained by analyzing 3 randomly selected wild-type (wt) PHEO (Table 1, [22]). To analyse the number of mitochondria, five cytoplasmic fields for each tumor were chosen at random and a count by point was performed [23].

### 2.6. Parents' Genetic Analysis

Genetic analysis was later extended to patient's mother and father who consented to be studied. After informed consent, genomic DNA was screened for directed research of the described *KIF1Bβ* mutations, using PCR and sequencing.

## 3. Results

### 3.1. Mutation Analyses

**Figure 1:**
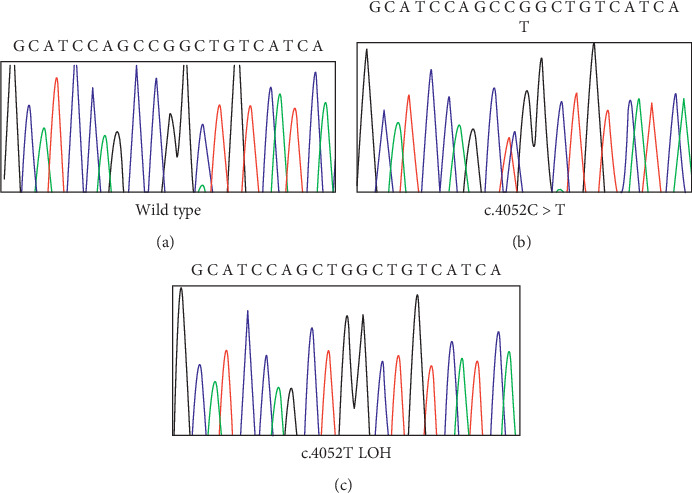
Wild-type (a) and patient electropherogram showing the germline mutation p.Pro1351Leu in exon 38 of the *KIF1Bβ* gene (b) and the somatic mutation showing the LOH (c).

NGS analysis of the 14 genes included in the panel revealed a novel germline, heterozygous missense mutation in the exon 38 of *KIF1Bβ* gene, causing the substitution of proline with leucine at position 1351 (c.4052C > T). The alteration was confirmed by Sanger sequencing and loss of heterozygosity (LOH) was detected in the tumor tissue (Figure 1, [24].

**Figure 2:**
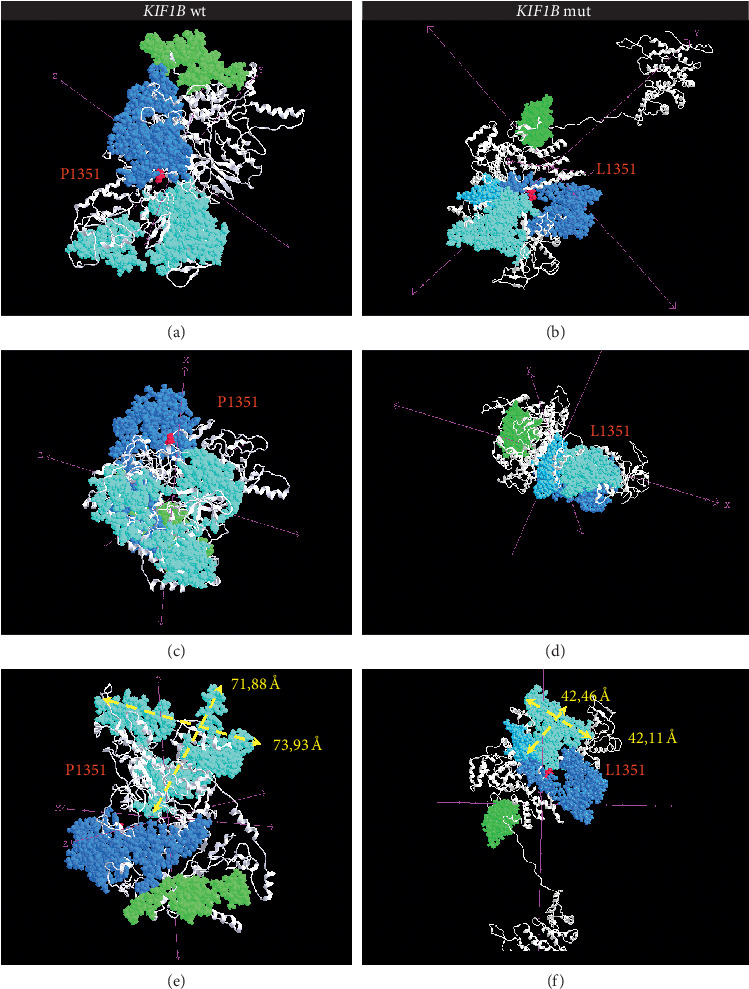
Predicted three-dimensional structures of *KIF1Bβ*, wild-type (a, c, e) and mutant proteins (b, d, f) observed at different angles. The point mutation P1351L was highlighted in red. In light blue, the two kinase domains 1B (aa 799–846; aa 899–928), in blue, DUF3694 domain (aa 1220–1366), and in green, the FHA domain (aa 512–581). The yellow arrows indicate the reduction of the length and width of the kinase domain (e, f).

The analysis of variance by querying different databases, i.e., Clinvar and Intervar, allowed us to classify the variant as uncertain (or unknown) significance. We subsequently assessed the pathogenic potential of novel transvertion using VarSome, a powerful suite which simultaneously queries the main tools of in silico analysis (Table 2, [25, 26]). Finally, the effect of the mutation P1351L on the structure of the protein was also examined. 3D modeling was generated from the aa 480 up to the C-terminal of the protein (aa 1770) using I-TASSER, and 91% of the residues were modeled at an accuracy of >90% (Figure 2, [27]). A comparison between wt *KIF1Bβ* protein and mutant protein P1351L revealed an overlap of two kinase domains 1B (aa 799–846; aa 899–928) with a reduction of surface of about 34% (light blue). In addition, a rotation of the predicted DUF3694 domain (aa 1220–1366) probably related to the Kinasic domain stabilization (blue) was observed. Moreover, a delocalization of the Forkhead-associated (FHA) domain (aa 512–581) (green), essential for the nuclear localization of the protein, was also observed. The results have been fully confirmed by gene ontology analysis derived from COACH. In particular, a possible cytosolic accumulation of the protein, a loss of microtubule function with consequent displacement of cytoplasmic organelles mainly of mitochondria, and a loss of cell motility during the formation of the central nervous system (CNS) were predicted.

### 3.2. Genetic Family Analysis

We found the same *KIF1Bβ* mutation in the father. He performed a CT scan and measurement of urinary metanephrines that were both negative.

### 3.3. Mitochondrial Status

Since *KIF1Bβ* is involved in the regulation of mitochondrial fission and apoptosis [8, 9] we assessed in tumor specimens the number and ultrastructure of mitochondria by electron microscopy as well as the proliferative index.

**Figure 3:**
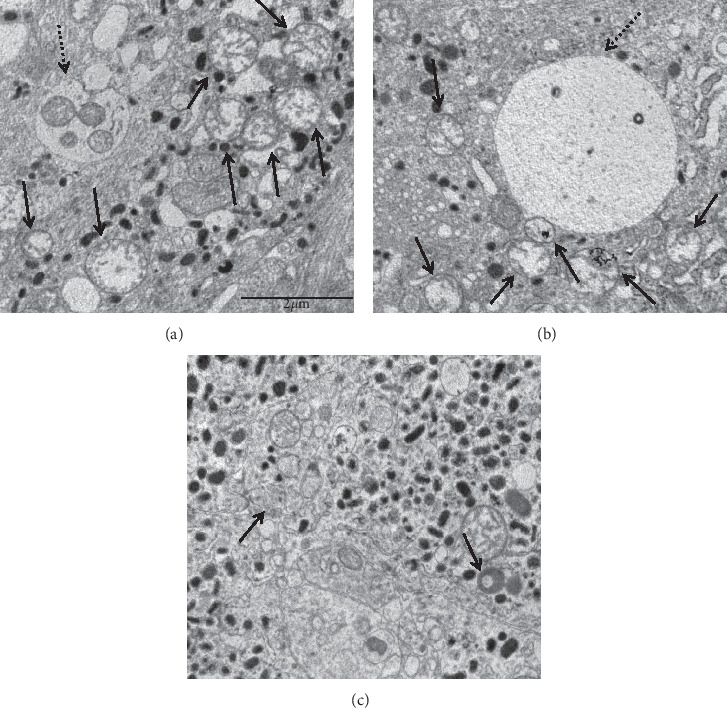
Representative ultrastructural micrographs of a pheochromocytoma bearing the *KIF1Bβ* mutation (a, b) in comparison with a wild-type tumor (c). Gene mutation is associated with the occurrence of numerous swollen mitochondria (dashed arrows) with reduction of cristae and matrix clearing. Several autophagic vacuoles (black arrows) containing remnants of organelles, including mitochondria (a) can also be seen. Magnification: x12,000.

Ultrastructural examination of the tumor specimens bearing the *KIF1Bβ* gene mutation revealed the presence of several swollen mitochondria with disrupted cristae and cleared matrix. Numerous autophagic vacuoles were also observed, some of which contained mitochondrial remnants (Figures 3(a)–3(b), [28]). By comparison, such abnormalities were not observed in a specimen of wt tumors (Figure 3(c), [28]). Moreover, performing the count by point of the mitochondria, we observed that the number of these organelles was almost two folds higher in *KIF1Bβ*-mutated tumor than in the nonmutated pheochromocytoma specimens (20.5 ± 2.1 and 10.4 ± 2.0 mitochondria/field, respectively).

## 4. Discussion

### 4.1. Role of *KIF1Bβ*

*KIF1Bβ* gene variants have been associated to the development of neural and nonneural tumors [10]. Germline *KIF1Bβ* variants have been rarely reported in patients with PHEO, while somatic variants have been more frequently found in tumor tissues.

The pathogenic mechanisms by which *KIF1Bβ* mutations cause the occurrence of neural and nonneural neoplasms are only partially known. Global transcription analysis of *KIF1Bβ* -mutant PHEO revealed that these tumors are transcriptionally related to RET- and NF1-mutated PHEO (Cluster 2) but independent from SDH- and VHL-associated tumors (Cluster 1). Furthermore, *KIF1Bβ* -mutant tumors are uniquely enriched for pathways related to glutamate metabolism and the oxidative stress response [10]. Additionally, kinesin KIF1B*β* acts downstream to prolyl hydroxylase to induce apoptosis; therefore, germline alterations compromising *KIF1Bβ* functions allow certain neuronal progenitor cells to escape the developmental culling [8]. Not all the *KIF1Bβ*-related tumors are associated with LOH, thus suggesting haploinsufficiency or epigenetic silencing of the wt allele in some tumors [10].

### 4.2. New Likely Pathogenetic *KIF1Bβ* Mutation

The transvertion (A > T) identified is a novel mutation and, as in such cases, the question arises whether it has to be considered pathogenic or not. The patient's family history was negative for the presence of neural or nonneural neoplasms, so we performed several in silico studies to answer this question. The bioinformatic tools classified the variant as pathogenic. The finding of LOH in the tumor tissue strengthened this assumption, and the adrenergic biochemical characteristic [23] of our patient's tumor is in agreement with the cluster 2 genetic profile.

### 4.3. Family Screening

On the basis of these results, we asked the patient's parents to undergo genetic testing which we performed after their informed consent. The same *KIF1Bβ* mutation was detected in the father which resulted negative in the biochemical and imaging screening.

In view of the *KIF1Bβ* mutation rarity, the penetrance is unknown and a low one, explaining the father's negative clinical picture can only be hypothesized. A further explanation might reside in a gene maternal imprinting similar to that found for *SDHD* and *SDHAF2* [29, 30]. Indeed, in the manuscript by Yeh et al., the family pedigree may be consistent with a maternal imprinting [10]. Nonetheless, the father's clinical follow-up remains mandatory.

### 4.4. Patient's Clinical Course

At diagnosis the biochemical phenotype was in line with the cluster 2 secreting pattern. However, at proband's follow up, MNu was found normal while NMNu, although decreased, was still elevated, prompting us to perform additional studies to explain this unexpected result. The biochemical phenotype detected after surgery may reflect the extra-adrenal localization of the disease. MRI, ^123^I-MIBG scintigraphy, and ^18^F-DOPA-PET suggested the presence of a metastatic lymph-node responsible for the increase in NMNu. No additional lesions were detected. During the follow up, the lymph-node was found slightly shrunk while NMNu normalized very slowly. The patient, who at present is normotensive and without any symptom, will undergo a life-long follow up in view of his genetic status, looking for PHEO recurrence, metastatic disease development, or the occurrence of other *KIF1Bβ* -linked tumors. The reason for the NMNu normalization two years after surgery is difficult to explain; it might be due to a tumor dedifferentiation or to a tissue necrosis.

### 4.5. First Evaluation of Mitochondrial Features in *KIF1Bβ*-Mutated Pheochromocytoma

To investigate the consequences of this mutation at the cellular level, we performed a deep analysis of the mitochondrial status in the tumor, finding a significantly higher aberrant morphology and number compared with wt tissues. This result is in line with the finding that *KIF1Bβ* altered activity is involved not only in neurodegenerative diseases but also in mitochondrial morphological aberration-associated with some tumors [20]. Previous data have shown that *KIF1Bβ* plays a role in the regulation of mitochondrial apoptosis. In particular, low expression of *KIF1Bβ* could lead to a reduction of mitochondrial fission and of subsequent apoptosis [8, 9]. We here observed an increased number of mitochondria (almost two fold higher) and the presence of autophagic vacuoles in the mutated tumor as compared with wt tissues. Considering our results, it is reasonable to hypothesize that the mutation found in *KIF1Bβ* is associated with a decrease in the physiological mitochondrial fragmentation and with the permanence of aberrant nonfunctional mitochondria in autophagic vacuoles, finally resulting in a decrease in the mitochondrial-induced apoptosis which may support tumor progression. It is possible to speculate that, to restore the mitochondrial homeostasis, tumor cells might activate a compensatory mitochondria neoformation leading to the increased number of these organelles. Further studies are needed to corroborate this hypothesis. Nonetheless, the present results are in line with the previous studies [8, 9] and represent the first observation performed in mitochondria of *KIF1Bβ*-mutated tissue. The evaluation of CN, DRP1, and YME1L1 expression in future studies on mutated tissues could improve our understanding of the role of *KIF1Bβ* alterations in human diseases.

## 5. Conclusions

In conclusion, we report a novel *KIF1Bβ* variant in a patient with PHEO. Our study confirms the importance of using target NGS in the genetic analysis of cancer patients in order to screen all the main susceptibility genes. At the same time, it recalls the necessity of a strict collaboration between geneticists and clinicians for a correct interpretation of the results and application of personalized medicine in view of an appropriate management of these patients. For the first time, we analyzed the mitochondrial features in a *KIF1Bβ*-mutated tissue.

## Figures and Tables

**Table 1:** Clinical characteristics of patients with nonmutated pheochromocytoma used for electron microscopy analysis.

Wt tumors	Age at diagnosis (years)	Sex (F/M)	PHEO/PGL	Secretion (A/NA)	Metastatic (yes/no)
Wt1	39	F	Right PHEO	A	No
Wt2	69	F	Left PHEO	NA	No
Wt3	52	F	Right PHEO	NA	No

Wt: wild-type; F: female; M: male; A: adrenergic; NA: noradrenergic (according to Eisenhofer et al. 2011) [22, 23].

**Table 2:** DANN pathogenicity score resulting from the simultaneous query of different in silico prediction tools.

DANN *version 2014*	Score 0.9987		
MutationTaster dbNSFP *version 4.0*	Prediction disease causing	Accuracy 1	Converted rankscore 0.81
Mutation assessor dbNSFP *version 4.0*	Prediction medium	Score 2.635	Rankscore 0.7711
FATHMM-MKL dbNSFP *version 4.0*	Coding prediction damaging	Coding score 0.9891	Coding rankscore 0.8853
FATHMM-XF dbNSFP *version 4.0*	Coding prediction damaging	Coding score 0.8661	Coding rankscore 0.7854
LRT dbNSFP *version 4.0*	Prediction deleterious	Score 0	Converted rankscore 0.8433
DEOGEN2 dbNSFP *version 4.0*	Prediction damaging	Score 0.8068	Rankscore 0.9513
EIGEN dbNSFP *version 4.0*	Prediction pathogenic	Raw coding 0.8034	Raw coding rankscore 0.8632
EIGEN PC dbNSFP *version 4.0*	Prediction pathogenic	PC raw coding score 0.798	PC raw coding rankscore 0.8966
SIFT dbNSFP *version 4.0*	Prediction damaging	Score 0.032, 0.03	Converted rankscore 0.4539
SIFT4G dbNSFP *version 4.0*	Prediction damaging	Score 0.039, 0.04	Converted rankscore 0.5111
PROVEAN dbNSFP *version 4.0*	Prediction damaging	Score -8.16, -8.3	Converted rankscore 0.9698
REVEL dbNSFP *version 4.0*	Prediction pathogenic	Score 0.699	Rankscore 0.8928
PrimateAI dbNSFP *version 4.0*	Prediction damaging	Score 0.8779	Rankscore 0.9367
MetaSVM dbNSFP *version 4.0*	Prediction damaging	Score 0.186	Rankscore 0.8567
MetaLR dbNSFP *version 4.0*	Prediction damaging	Score 0.5961	Rankscore 0.8559
FATHMM dbNSFP *version 4.0*	Prediction tolerated	Score -0.9, -0.81, -0.89	Converted rankscore 0.7489
MVP dbNSFP *version 4.0*	Prediction uncertain	Score 0.7537	Rankscore 0.7514
MutPred dbNSFP *version 4.0*		Score 0.66	Rankscore 0.7978

The value range is 0 to 1, with 1 given to the variants predicted to be the most damaging (http://varsome.com/variant/hg19/kif1b%3Ac.4052C>T) (according to Quang et al. 2015, [26]).

## Data Availability

The data used to support the findings of this study are available from the corresponding author upon request.
